# Factoring in the Complexity of the Cystic Fibrosis Lung to Understand *Aspergillus fumigatus* and *Pseudomonas* *aeruginosa* Interactions

**DOI:** 10.3390/pathogens9080639

**Published:** 2020-08-06

**Authors:** Emily Beswick, Jorge Amich, Sara Gago

**Affiliations:** 1 Manchester Fungal Infection Group, Faculty of Biology, Medicine and Health, The University of Manchester, Manchester Academic Health Science Centre, Core Technology Facility, Grafton Street, Manchester M13 9NT, UK; 2 Academic Unit of Medical Education, Medical School, University of Sheffield, Beech Hill Road, Broomhall, Sheffield S10 2TG, UK; efbeswick1@sheffield.ac.uk

**Keywords:** *Aspergillus fumigatus*, *Pseudomonas aeruginosa*, cystic fibrosis, combined infection

## Abstract

*Pseudomonas aeruginosa* has long been established as the most prevalent respiratory pathogen in Cystic Fibrosis (CF) patients, with opportunistic infection causing profound morbidity and mortality. Recently, *Aspergillus fumigatus* has also been recognised as a key contributor to CF lung deterioration, being consistently associated with decreased lung function and worsened prognosis in these patients. As clinical evidence for the common occurrence of combined infection with these two pathogens increases, research into the mechanism and consequences of their interaction is becoming more relevant. Clinical evidence suggests a synergistic effect of combined infection, which translates into a poorer prognosis for the patients. In vitro results from the laboratory have identified a variety of possible synergistic and antagonistic interactions between *A. fumigatus* and *P. aeruginosa*. Here, we present a comprehensive overview of the complex environment of the CF lung and discuss how it needs to be considered to determine the exact molecular interactions that *A. fumigatus* and *P. aeruginosa* undergo during combined infection and their effects on the host.

## 1. Cystic Fibrosis

Cystic fibrosis (CF) is a life-limiting, inherited autosomal recessive disorder resulting from the presence of one or more disease-causing mutations in the cystic fibrosis transmembrane conductance regulator (CFTR) gene. The global prevalence of CF is approximately 70,000–100,000 and within the United Kingdom (UK) alone, over 10,000 people are living with this disease [1,2]. The incidence of CF differs between geographical locations and ethnic groups; however, the highest rate is amongst Caucasian populations, especially those of northern European descent, in whom incidence is 1 in between 2500–4000 live births [1,3].

Since CF was first described over 80 years ago [4], patient life expectancy has vastly improved. In the 1950s, median life expectancy for a new-born diagnosed with CF was as little as several months and the main causes of death were meconium ileus or malnutrition due to pancreatic failure [5]. Life has dramatically improved for CF patients due to advances in modern medicine such as the development of mucolytic agents, specific anti-pseudomonal drugs, pancreatic enzyme replacement therapy as well as the introduction of a multi-disciplinary approach to CF patient care [5,6,7]. Yet despite these improvements, patients continue to deal with intensive treatment regimens and frequent hospitalisations that impact on their quality of life. Additionally, profound morbidity largely due to recurrent lung infections and progressive deterioration in lung function [8] makes progressive pulmonary disease resulting in fatal respiratory failure the main cause of death in CF patients [5,9].

The CFTR gene itself is located on the long arm of chromosome 7 at position 31.2 [10] and is subjected to a complex and strict transcriptional control that is not fully understood yet [11]. CFTR encodes a unique cyclic adenosine monophosphate (cAMP)-dependent ion channel, which is the only known member of the ATP-binding cassette superfamily that does not function as an active transporter [3]. The protein comprises five domains: (i) two pseudo-symmetrical membrane-spanning domains (MSDs) each made up of six helices connected by intra- and extra-cellular loops, (ii) two highly conserved nucleotide-binding domains (NBD1 and NBD2) and (iii) a regulatory domain characterised by charged residues and multiple consensus phosphorylation sites [12].

CFTR is located at the apical surface of epithelial cells, where it maintains ion transport across exocrine, endocrine and pulmonary epithelial interfaces [3]. CFTR regulates sodium [13,14], potassium [15,16,17], sodium bicarbonate [18], outward-rectifying chloride channels [19] and calcium-activated chloride channels [20,21]. CFTR also regulates ATP release, vesicle trafficking and cytokine expression [3].

More than 2000 variants of the CFTR gene have been identified [22], many of which are associated with causation of CF disease [5,23]. The majority of mutations identified in CFTR are localised to NBD1. Relatively few disease-causing mutations have been confirmed in NBD2 [24].

Mutations are classified into six groups according to their effect on protein expression and physiological function (Table 1) [25]. Although the mechanism and extent of depletion may differ, all classes of CFTR mutation result in overall functional loss of the protein in target cells [26].

The most common CF-causing mutation is a class II mutation resulting in deletion of phenylalanine at amino acid 508 (ΔF508). Up to 90% of CF patients are homozygous for ΔF508 and this deletion constitutes up to two-thirds of mutated alleles in northern Europe and North America [27,28]. F508 is located in a highly conserved, alpha-helical region (residues 495–565) in NBD1 [29]. Deletion disrupts ATP-dependent interactions in the F1-ATPase domain of NBD1 and alters kinetic and thermal properties [12,30]. These changes affect protein assembly and post-translational stability such that maturation of core glycosylated CFTR^ΔF508^ to complex glycosylated fails, leading to endoplasmic retention of CFTR^ΔF508^ [31]. As a result, CFTR^ΔF508^ is never trafficked to the cell membrane and the concentration of functional CFTR in target cells is minimal. 

## 2. Clinical Manifestations of Cystic Fibrosis

Functional loss of the CFTR ion channel leads to an inability to maintain osmotic balance across all epithelial surfaces, resulting in wide-ranging pathophysiological effects that cause multi-system organ dysfunction [32]. Extra-pulmonary manifestations of mutant CFTR are intestinal obstruction (including meconium ileus in new-borns), exocrine pancreatic insufficiency, biliary cirrhosis of the liver, abnormal growth and infertility in males [33,34]. In the long-term, patients may develop cystic-fibrosis related diabetes mellitus [35] and are at increased risk of clinical depression and anxiety [34]. However, the major pathological consequence of CF is chronic lung disease. 

Despite the monogenic nature of CF disease, it is likely that clinical manifestations are the product of multiple pathophysiological changes [36]. This new appreciation of CF as a multi-factorial disease may also explain the failure of mucolytic agents or aerosolised bicarbonate alone to rescue disease status in animal models, as addressing a singular aspect of the pathology is insufficient for cure [1,5,37]. There are two main hypotheses proposed to explain the pathogenesis of CF lung, the “low-volume hypothesis” and the “pH hypothesis”.

The “low-volume hypothesis” is the most well-established theory for the pathogenesis of CF pulmonary disease [33]. This hypothesis stipulates that in CF lungs, loss of chloride efflux and excess sodium and water reabsorption causes dehydration of both the airways surface liquid (ASL) and mucus secretions. Cilia are rapidly compressed by the mucus layer [27], resulting in pulmonary ciliary dyskinesis and impaired mucociliary clearance [3]. Thus, stagnant mucus plaques form in CF airways, providing microaerobic and even anaerobic conditions that facilitate colonisation and persistence of various fungal and bacterial pathogens, notably *P. aeruginosa* [38].

An alternative hypothesis is based upon alteration of the ASL pH: loss of function in CFTR impairs bicarbonate transport across the pulmonary epithelia and reduces the ASL pH [39]. This directly inhibits the innate antimicrobial activity of the many antimicrobial molecules contained in ASL including lactoferrin, lysozyme, beta-defensins, LL-37, secretory leukocyte peptidase inhibitor and surfactant protein A and D [40]. This hypothesis is supported by investigations in a porcine model, where it was shown that both the individual and synergistic activities of these antimicrobial molecules were significantly reduced in CF piglets, compared to wild type [39]. Furthermore, administration of aerosolised sodium bicarbonate to increase ASL pH was found to rescue bacterial permeabilisation and killing mechanisms in CF pigs [39]. Elimination of CFTR-regulated bicarbonate secretion lowers not only ASL pH, but the pH of the submucosal glands also [41]. Submucosal glands produce mucus strands that detach from the epithelium and comprise the mucus layer. Lack of mucus strand detachment thus contributes to abnormal mucus composition, impaired mucociliary transport and the obstruction of small airways [42,43]. 

In addition to defects in airway epithelium, mutant CFTR also gives rise to immune system defects in CF airways. In particular, the role of the neutrophil is well characterised, with neutrophil-mediated inflammation identified as a key driver of lung damage in CF [44]. Chronic colonisation continuously activates pathogen recognition receptors, causing excessive influx of polymorphonuclear neutrophils (PMNs) into the lungs [33]. However, mutant CFTR impairs degranulation and phagocytosis, so neutrophils cannot kill pathogens effectively. Neutrophils also have enhanced lifespan in CF due to dysregulation of apoptotic pathways and accumulate within the airways causing oxidative stress, which further activates inflammatory pathways and IL-8-mediated PMN recruitment [45]. Neutrophils also release elastase, a powerful serine protease that drives bronchiectasis by digesting cellular elastin and secondly, increases mucus production by cleavage of epithelial cell-surface sodium channels [46]. The result is reduced pathogen clearance, chronic inflammation and damage to lung tissue [33].

The host response to infection is compromised in CF; however, abnormally elevated levels of the pro-inflammatory cytokines IL-6, IL-1β, IL-8 and TNFα have also been found in uninfected CF immortalised cell lines and ex vivo lung explants [27,47,48]. These findings have led to the hypothesis that mutated CFTR also makes epithelial cells intrinsically more inflammatory, and that the primary defect in CF may be a dysregulated host inflammatory response [27]. In addition, the production of anti-inflammatory substances like IL-10, docosahexaenoic acid and lipoxin [49,50] is reduced in CF, resulting in a cytokine imbalance that favours a pro-inflammatory lung environment [8,51]. Indeed, T helper cell type (Th)-2, Th-17 and T-regulatory cell responses are also dysregulated at numerous levels [52]. A plausible interpretation of these findings by Elborn (2016), is that intrinsic pro-inflammatory cell properties may b the primary cause of inflammation in the CF lung during infancy; however exposure to pathogenic microorganisms and ineffective immune response becomes the main driver of chronic inflammation in later life [5].

In consideration of the evidence, the CF lung may be described as a pro-inflammatory environment characterised by viscous mucus secretions, acidic ASL pH, and impaired innate and adaptive immune responses to pathogen challenge. Overall, these pathophysiological changes contribute to the formation of a permissive pulmonary niche, facilitating chronic lung colonisation with opportunistic microorganisms. Thus, CF patients are vulnerable to recurrent cycles of infection and inflammation, inevitably leading to pulmonary system failure [53].

## 3. Dysbiosis of the Pulmonary Microbiome in Cystic Fibrosis

The lung was, until recently, thought to be a sterile organ (unless infected). Previously, standard microbial culture failed to replicate the environment inside the lung sufficiently to enable bacterial growth from bronchoalveolar lavage (BAL) samples, perpetuating the sterile lung theory. The advent of molecular techniques has since generated evidence to demonstrate that healthy lungs are colonised by a distinct bacterial community [54], which is putatively implicated in the pathology of chronic lung diseases like CF [55]. 

In a healthy host, the most prevalent phyla are *Firmicutes* and *Bacteroidetes* [54] with lesser numbers of *Proteobacteria* and *Actinobacteria* also present [56]. The core pulmonary microbiota comprises: *Pseudomonas*, *Streptococcus*, *Haemophilus*, *Neisseria*, *Prevotella*, *Fusobacterium* and *Veillonella* species [57]. Growth conditions in the lung are determined by nutrient and oxygen availability, pH, temperature, as well as the host inflammatory response. In health, these three factors are tightly regulated, but in patients with chronic lung diseases like CF, they are imbalanced by compromise of host defences [54]. Research undertaken to characterise the microbiota in chronic lung diseases has identified aberrant outgrowth of one of the four main phyla [56] leading to dysbiosis of the microbiome. For CF, *Proteobacteria* and *Actinobacteria* are overrepresented, correlating with clinical evidence of bacterial infections that predominate in these patients [56,58].

CF lung pathogens are typically opportunistic, environmental bacteria and fungi that rarely cause disease in healthy hosts. As discussed above, CF patients fail to clear invading bacteria and fungi, which facilitates chronic infection. Chronic colonisation and infection results in the production of microbial toxins, proteases and invasive growth, e.g., fungal hyphae, which causes inflammation, epithelial tissue damage and overall decline in pulmonary function. Microbial growth within the lung also triggers pro-inflammatory pathways and leukocyte recruitment, which further damages the epithelia and induces secretion of viscous mucus [33]. The respiratory “virome” remains largely unknown [59,60]. Few studies have investigated the contribution of viruses to the CF lung metagenome, mainly due to the current lack of consensual viral gene markers comparable to the 16S ribosomal system in bacteria [61,62]. Despite this incomplete picture, it remains that disease course and severity is increased when CF patients contract viral pneumonias [63].

The CF microbiome is dynamic and undergoes compositional changes as pulmonary disease progresses. However, relating these to pulmonary exacerbations has proved challenging [59]. Cross-sectional and longitudinal studies have demonstrated that CF patients are colonised by increasing numbers of microorganisms as they age, with good airway diversity typically maintained until adolescence [59]. The most common microorganism found in the airways of CF children is *Staphylococcus aureus*, which is substituted for by *P. aeruginosa* as patients transition to adulthood [2,64,65]. The relationship between these two pathogens is not yet understood, but some clinical studies have indirectly supported an inverse association between them [66,67]. However, it seems to be clear that the development of chronic infections caused by *P. aeruginosa* are associated with lung function decline and thus treatments aiming to eradicate initial infections are often recommended [68,69,70]. However, the administration of cumulative antibiotic courses to treat recurrent lung infection introduces selective pressure that favours survival of resistant bacteria [59,71]. 

Data from both the UK and US 2018 Cystic Fibrosis Trust patient registry reports [2,65] show that *P. aeruginosa* overall remains the most prevalent pathogen colonising CF lungs due to its capacity to specifically adapt to the CF airway and grow as biofilm, discussed in detail in the next section. Other important pathogens known to colonise and cause infection in CF patients include: *Haemophilus influenzae, S. aureus* and meticillin-resistant *S. aureus*, *Stenotrophomonas maltophilia,* the *Burkholderia cepacia* complex [59] and atypical non-tuberculous *Mycobacteria* species [72]. Anaerobes are also abundant in CF sputum and BAL samples [73], however their contribution to pulmonary morbidity remains unclear [68]. Anaerobes likely contribute to the “resistome” (the total number of antibiotic-resistance genes carried by microorganisms in the lungs) but whether their overall effect is beneficial, or detrimental remains to be deciphered [73,74].

Finally, the CF mycobiome has recently emerged as a promising area of research. Previously, the mycobiome was under-recognised due to the low sensitivity of culture-based detection methods, combined with low overall numbers of fungi in the lungs (in comparison to bacteria) and the general perception of these as environmental contaminants [75,76]. In fact, the CF mycobiome is fairly diverse: *Aspergillus*, *Candida*, *Cladosporium, Penicillium*, *Scedosporium* and *Exophiala* species have all been identified in CF cohorts [77,78]. The contribution of *A. fumigatus* to pulmonary disease in CF is of particular interest, as this pathogenic fungus is frequently isolated from patient respiratory samples and has been associated with worsened prognosis and pulmonary function [79]. 

Herein, the individual contribution of *P. aeruginosa* and *A. fumigatus* to CF lung disease will be discussed, before reviewing the current state of the field regarding combined infection with these two classic CF pathogens.

## 4. *Pseudomonas aeruginosa* Is the Predominant Respiratory Bacterial Pathogen in Cystic Fibrosis

*P. aeruginosa* is a Gram-negative bacterium and facultative anaerobe, found ubiquitously in soil and water. *P. aeruginosa* is a prolific pathogen that infects many different tissues, causing serious skin and soft tissue infections; endocarditis; urinary tract infections; gastrointestinal infections; meningitis; ocular and ear infections; and ventilator-associated pneumonias [33]. *P. aeruginosa* causes both chronic and acute infections, the majority of which occur in the immunocompromised, hospitalised patients or those with an underlying pulmonary disease, like CF.

*P. aeruginosa* has long been established as a formidable, opportunistic pathogen in CF patients, with up to 80% of individuals chronically colonised by age 20 [80]. The contribution of this bacterium to progressive lung disease in CF is so profound, that *P. aeruginosa* colonisation status remains the single most important determinant of morbidity and mortality in CF [3]. Despite intensive antibiotic therapy and the development of drugs with enhanced anti-pseudomonal activity, the vast majority of CF patients that experience intermittent infection with *P. aeruginosa* will go on to establish chronic infection (defined as infection for >6 months +/-positive serology) [81].

Patients are periodically colonised by phylogenetically distinct strains of *P. aeruginosa* from early on in life and longitudinal studies have demonstrated that by age three, 98% of CF patients have positive serology and/or culture for *P. aeruginosa* [81,82]. During this intermittent stage, infection is caused by planktonic *P. aeruginosa* cells and may still be successfully eradicated by inhaled antibiotic therapy [81]. Transition from intermittent to chronic infection represents successful adaption of the bacterium to CF airways, and is a seminal clinical event that precipitates pulmonary decline and increased mortality [83].

Adherence to the lung epithelium is mediated by *P. aeruginosa* flagella and pili [33]. Once *P. aeruginosa* has colonised the epithelium, diffusible LasR and RhiR quorum sensing molecules regulate the expression of immunomodulatory virulence factors to facilitate survival in the hyperinflammatory, neutrophil-rich CF lung [84]. For example, *P. aeruginosa* secretes exotoxins via its Type III secretion system that can directly lyse neutrophils [85], pyocyanin induces neutrophil apoptosis [86] and alkaline protease degrades complement proteins to evade opsonophagocytosis [87].

Crucially, *P. aeruginosa* possesses a highly plastic genome that undergoes micro-evolution within the host, allowing specific adaption to the CF niche and rapid acquisition of gene elements encoding multi-drug resistance determinants [3]. Evolution is driven by the uniquely stressful environment that *P. aeruginosa* encounters in CF lungs, for example high antimicrobial drug concentration and neutrophil abundance [33]. Micro-evolutions arise by spontaneous mutation and subsequent selection of phenotypic/genotypic variants with enhanced survival [88]. The emergence of variants within an infected individual facilitates transition to chronic infection [89]. Common variant characteristics include downregulation of immunostimulatory virulence factors [90], overexpression of exopolysaccharides [81] and decreased motility to promote biofilm formation [91,92,93,94]. In fact, biofilm development is characteristic of chronic infections, which are virtually impossible to eradicate as biofilms confer increased resistance to antimicrobials and immune killing [95,96]. One of the most significant variants that has been associated with biofilm formation and establishment of chronic infection is the emergence of mucoid colonies [91]. Furthermore, mucoid colonies are strongly associated with severe bronchiectasis and rapid respiratory decline in CF patients [97]. Mutations in the *mucA* gene give rise to this phenotype, which overexpresses alginate exopolysaccharide. Mucoidy can inhibit complement activation [98]; is resistant to neutrophil extracellular traps [99]; evades phagocytic killing [100]; resist innate antimicrobial peptides including LL-37 [101]; and is recalcitrant to even specifically anti-pseudomonal drugs [33,102]. 

## 5. *Aspergillus fumigatus* Is the Predominant Respiratory Mould Pathogen in Cystic Fibrosis

*Aspergilli* are filamentous, saprophytic fungi that decompose organic matter and are integral to the recycling of carbon and nitrogen compounds in numerous environmental niches [103]. Various characteristics allow *Aspergillus* species to survive within physiologically harsh environments, including rapid germination, growth within a wide pH range and at high temperatures (<60 °C) and flexible nutrient metabolism [104]. *Aspergilli* are therefore found worldwide in soil, decaying vegetation and ambient air as well as in artificial environments such as composting facilities, air handling systems and building insulation [103,105]. Human exposure to these ubiquitous fungi is inescapable; however, colonisation, infection and disease only occurs if host defences fail to eradicate fungal fragments or spores (conidia) from the body [106]. Disease is therefore a product of complex interplay between fungal virulence factors and host-related factors such as immune status and pulmonary structure [107,108]. 

The highest burden of aspergillosis is within the pulmonary system [109] because exposure to infectious particles most frequently occurs via inhalation of airborne conidia, fungal allergens or other cell fragments. *Aspergillus* sporulate abundantly and a single colony can generate several billion conidia, which are then disseminated by air and other routes [110]. Humans inhale many litres of air every minute, resulting in constant exposure to thousands of airborne conidia [106,111]. Thus, respiratory forms of aspergillosis are prevalent in the global population: chronic pulmonary aspergillosis affects 3 million people; fungal allergy including sensitisation over 10 million; and invasive diseases affect 0.2 million [106,109]. 

*A. fumigatus* is the most pathogenic species within the genus as a whole, although *A. flavus, A. nidulans*, *A. niger*, and *A. terreus* are capable of causing disease in humans [110]. In addition, *A. fumigatus* is associated with highest morbidity in CF patients [75]. The success of *A. fumigatus* as a respiratory pathogen is multifactorial; however, a critical factor is that its spores are small enough (2–3 µm diameter) to penetrate deep into the lungs and reach the distal alveoli [110,112]. 

Aspergillosis rarely affects healthy hosts because deposited spores are rapidly eradicated by innate defences. The first line of defence is the lung epithelium itself, where inhaled conidia and fungal fragments encounter mucopolysaccharide secretions in the upper respiratory tract that effectively trap and clear conidia via the mucociliary pathway. However, *Aspergillus* spores penetrating as far as the alveoli bypass this defence mechanism. Specialised epithelial cells also secrete antimicrobial molecules and recruit immune cells to the deposition site [106]. In order for invading pathogens to be cleared efficiently, lung epithelial cells, macrophages and dendritic cells maintain the balance between Th-1, Th-17, Th-2 and T-regulatory cells. Th-1 cell proliferation is critical in responding to fungal challenge as this pathway activates neutrophils, T-cells and macrophages, which phagocytose fungal cells [106,112]. The role of neutrophils in *A. fumigatus* challenge is well defined; these cells phagocytose conidia [110] and employ extracellular traps (NETs) to inhibit hyphal growth too large for phagocytosis [113].

In CF, these innate host defences are impaired and *Aspergillus* conidia germinate. Pulmonary aspergillosis may manifest as four distinct disease entities in CF patients: chronic colonisation, *Aspergillus* hypersensitisation, allergic bronchopulmonary aspergillosis (ABPA) or *Aspergillus* bronchitis [79]. Two different models have been proposed to explain development of aspergillosis in CF, each supported by limited clinical research. The first proposes sequential disease progression with increasing severity, for example developing *Aspergillus* bronchitis subsequent to persistent colonisation, whereas the other considers each disease entity to be separate [79].

*Aspergillus* colonisation prevalence in CF patients is between 10–57%, with higher prevalence being associated with increasing age and poorer lung function [79]. Colonisation occurs when *Aspergillus* spores germinate and form mycelium at the epithelial surface [76]. The pathophysiology of progression from colonisation to active pulmonary infection has not yet been fully determined, however, chronic colonisation is a predecessor to pulmonary aspergillosis in compromised hosts [106,107].

Bronchial colonisation with *A. fumigatus* certainly drives the development of allergic forms of aspergillosis [76,114]. *Aspergillus* hypersensitisation is characterised by a raised immunoglobulin E response to *Aspergillus* allergens without clinical symptoms [76,107]. Baxter et al. (2013) also showed that hypersensitisation is distinguishable from ABPA by negative galactomannan test and lack of raised serum IgG [115]. 

ABPA is now a well-defined clinical entity in CF patients, despite challenges in the field regarding standardisation of diagnostic criteria [116]. This allergic aspergillosis is characterised by an exaggerated Th2-mediated immunoglobulin E response to *Aspergillus* allergens, with eosinophilia and mast cell degranulation [76,107]. Prevalence of ABPA in CF patients varies between 1–18% depending on geographical region but is likely to be underdiagnosed [79]. Classic manifestations of ABPA are respiratory exacerbation (wheeze), positive serology against *Aspergillus* (IgE) and visible pulmonary infiltrates upon imaging [117]. 

*Aspergillus* bronchitis is an emerging form of clinical aspergillosis in CF patients, affecting approximately 9% of this cohort [118]. Pseudomembranous mycelial growth in the airways causes breathlessness, recurrent respiratory infections (that respond poorly to antibiotics) and mucus plugging. Bronchoscopic examination typically reveals bronchial erythema, ulceration and superficial hyphal invasion of the mucosa [106,119]. 

The contribution of *A. fumigatus* to chronic pulmonary disease in CF is now clear and well-recognised. Advances in next-generation sequencing and culture-independent techniques have made the study of diverse microbial communities possible. Now, inter-kingdom interactions between *A. fumigatus* and dominant bacterial pathogens like *P. aeruginosa* have become established as a promising area of research due to their clinical implications. 

## 6. Combined Infection with *Aspergillus fumigatus* and *Pseudomonas aeruginosa* in Cystic Fibrosis

*A. fumigatus* and *P. aeruginosa* are the most prevalent fungal and bacterial species that infect CF airways, respectively [120]. *P. aeruginosa* has long been recognised as a key pathogenic member of the CF lung microbiota; however, until recently, the contribution of *A. fumigatus* to pulmonary disease in CF was underestimated. Application of molecular techniques such as next-generation sequencing has facilitated characterisation of the complex microbial community that colonises CF lungs, bringing about a paradigm shift in the management of polymicrobial lung infections in this patient cohort [83]. 

Clinical evidence has for some time suggested high frequency of *A. fumigatus-P. aeruginosa* combined infection in CF patients. Both pathogens have been isolated simultaneously from patient sputum samples, with *A. fumigatus* identified in up to 60% of patients with chronic *P. aeruginosa* and conversely, *P. aeruginosa* identified in up to 64.2% of patients positive for *A. fumigatus* [121,122]. Indeed, it has been reported that *P. aeruginosa* infection is associated with higher incidence of concurrent and subsequent *A. fumigatus* infection [123], and that *A. fumigatus* colonisation was associated with an increased risk of *P. aeruginosa* colonisation [124]. A recent systematic review and meta-analysis estimated pooled combined infection prevalence to be ~15.8%, with a range of 2.2–44.8% [120]. For these patients, persistent combined infection with *A. fumigatus* and *P. aeruginosa* is associated with overall worsened lung function [125]. Unfortunately, studies investigating clinical manifestations of *A. fumigatus-P. aeruginosa* combined infection in CF patients are limited and prospective longitudinal studies are required in order to confirm these associations [83].

In vitro studies have mostly described antagonistic interactions between these two pathogens. For example, *P. aeruginosa* is known to inhibit *A. fumigatus* through the action of several secreted molecules: secretion of dirhamnolipids inhibit growth by blocking β1,3 glucan synthase [126], homoserine lactones suppress hyphal growth [127] and reactive oxygen species (ROS)-producing phenazine compounds cause fungal death [128]. Although paradoxically, promotion of *A. fumigatus* by *P. aeruginosa* also occurs through phenazine secretion, whereby sub-bacteriostatic concentrations of phenazine induce iron uptake by *A. fumigatus* [126,129]. *P. aeruginosa* also mediates *A. fumigatus* growth inhibition by iron starvation through various mechanisms including iron chelation by pyoverdine [130,131] and pyocheline (a compound that also triggers ROS production) [132]. In addition, the Pf4 class of bacteriophages produced by *P. aeruginosa* are known to bind to and inhibit the metabolic activity of *A. fumigatus* biofilms [133]. With regards to *A. fumigatus* inhibition of *P. aeruginosa*, the main inhibitory agent secreted by the fungus is gliotoxin, which is a broad-spectrum inhibitor of bacterial biofilm formation [134]. Synergistic interactions have also been described in vitro; for example, *P. aeruginosa* can indirectly stimulate the growth of *A. fumigatus* by release of a volatile organic compound, dimethyl sulphide, demonstrating that *A. fumigatus* and *P. aeruginosa* interact even at distance [135]. Additionally, it has been described that the *A. fumigatus* stress response to pseudomonal phenazines can degrade those molecules into chemicals that induce *Aspergillus* siderophore and enhance fungal iron scavenging [136,137]. *A. fumigatus* response to iron is also modulated by a pseudomonal quorum sensing molecule, *P. aeruginosa* quinolone signal, which enhances fungal growth and metabolism under iron-rich conditions, similar to those found in the CF lung environment [138]. Conversely, recent proteomic analysis by Margalit et al. has shown that *A. fumigatus* can confer fitness to *P. aeruginosa* by increasing environmental nutrient availability, especially of amino acids and nitrate, which thereby stimulates bacterial growth and pathogenicity [139]. The nature of *A. fumigatus-P. aeruginosa* interactions becomes more complicated when other factors are considered. For instance, the inhibitory effect of *P. aeruginosa* on *A. fumigatus* is reduced under hypoxia, which is the actual environment where these pathogens interact [140]. Furthermore, small colony variants of *P. aeruginosa* with high-level pyoverdine expression (mucoidy phenotype), which are commonly isolated from chronically colonised CF lungs, have been shown to be more inhibitory towards *A. fumigatus* [141]. Inhibitory capacity is also determined by mode of growth, as *P. aeruginosa* is more inhibitory towards *A. fumigatus* in biofilm form, compared to planktonic [129] Moreover, *P. aeruginosa* is more inhibitory when growing as a biofilm compared to planktonic, but is only weakly inhibitory towards mature *A. fumigatus* biofilms [142]. In context, the majority of CF patients are chronically colonised by *P. aeruginosa* prior to *A. fumigatus* and therefore interactions would most likely occur between pre-formed *P. aeruginosa* biofilm and desegregated hyphal, rather than thick mycelial, fungal growth [129]. 

In vitro results reflect that both synergistic and antagonistic interactions are possible between *P. aeruginosa* and *A. fumigatus*. Differences in the type of interaction detected in some of the studies described above, is likely due to the experimental set up and methodology. For example, interactions will be dependent on the medium used to grow the microorganisms, as the nutrient environment strongly influences the production of metabolites that can exert an effect on the other microbe (e.g., the siderophores, pyoverdine and pyochelin, are produced only under iron limiting conditions). In any case, both synergistic and antagonistic interactions may have a deleterious effect on the host. Herein, synergism can cause increased pathogen growth and antagonism can result in increased damage to host tissues due to production of toxic molecules. An important first step to clarify the specific relationship that *A. fumigatus* and *P. aeruginosa* establish in the human lung, would be to consider the contribution of the host and host–pathogen interactions. Thus far, only a handful of studies have investigated the pathogen–pathogen interaction including either host cells or an in vivo model. Reece et al. reported a mutually antagonistic effect that may increase the pro-inflammatory response in CF epithelial cells and resulted in *P. aeruginosa* causing increased mortality in *Galleria mellonella* after pre-exposure to a non-lethal dose of *A. fumigatus* [134]. Similarly, we recently described that combined infection triggered increased fungal and bacterial burdens and caused greater mortality in *G. mellonella* [143]. Margalit and colleagues showed that *A. fumigatus* renders human alveolar epithelial cells unable to internalise *P. aeruginosa*, which resulted in higher proliferation of the bacteria in combined infection, compared to in single infection [144]. In addition, Briard et al. found that sequential infection of monocytes by *P. aeruginosa* and *A. fumigatus* synergistically increased the secretion of the pro-inflammatory cytokine IL1β, which could cause an over-inflammatory environment in patients with combined infection [132]. Finally, Smith et al. have reported that *P. aeruginosa* production of cytotoxic elastase is enhanced in the presence of *A. fumigatus*, which caused more damage to epithelial cells [145]. Overall, these studies suggest that combined infection causes greater damage to the host tissues, independent of the sequence of infection.

In summary, it will only be possible to determine the real nature and exact interactions that *A. fumigatus* and *P. aeruginosa* undergo during combined infection, when the complexity of the CF lung is factored into experimental design. For this purpose, animal models seem key; however, to date, only two studies have attempted to model pulmonary combined infection in mice, with limited results. The first modelled combined infection by chronically infecting corticosteroid-treated mice with *A. fumigatus* using agar beads embedded with conidia, then twelve days later superinfecting those mice with *P. aeruginosa* [146]. In these superinfected mice, Yonezawa et al. found no change in fungal burden and increased bacterial burden, with statistical analysis showing a non-significant decrease in mortality compared to single *A. fumigatus*-infected mice [146]. These results diverge from the current understanding amongst clinicians, which is that combined infection increases patient mortality. In fact, the scope and findings of this study were limited by its methodology in several different aspects. Firstly, the reverse sequence of *A. fumigatus* superinfection on top of chronic *P. aeruginosa* infection was not assessed, despite the majority of CF patients being chronically colonised by *P. aeruginosa* prior to *A. fumigatus* [129]. Secondly, the inoculation of conidia embedded into potato dextrose agar beads does not represent a natural infection process, as fungal metabolism is affected by the rich medium, and thus methodology may have led to artefactual interactions in this model. Finally, corticosteroid treatment is not an adequate method for modelling chronic lung inflammation in mice. 

The same group attempted to assess combined infection and evaluate antimicrobial drug efficacy in vivo using an alternative methodology [147]. In this study, mice were simultaneously inoculated by nasal administration with an *A. fumigatus* conidial suspension and *P. aeruginosa* cell suspension. Unfortunately, the use of a leukopenic model resulted in combined infected mice dying within three days post-infection, which impeded any assessment of the *A. fumigatu-P. aeruginosa* interaction [147]. These two studies highlight the technical problems currently encountered in an in vivo combined infection model, where animals face high pathogen challenge and experimental design is complicated. 

In conclusion, the complexity of described interactions between these two pathogens indicates that the nature of their relationship, as well as their combined effects on the host, is strongly dependent on the specific conditions in which they are found. The CF lung is extremely complex and therefore, this unique environment needs to be factored in to be able to determine the exact interactions that *P. aeruginosa* and *A. fumigatus* undergo during combined infection.

## 7. Future Directions for Clarifying *A. fumigatus-P. aeruginosa* Interactions in CF

**Figure 1:**
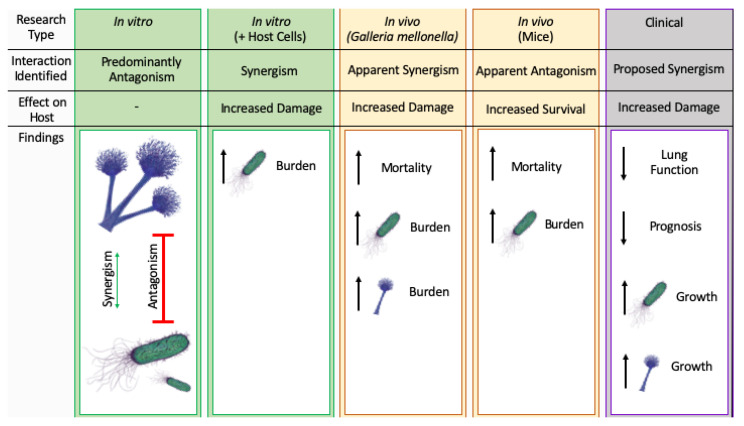
*Pseudomonas aeruginosa*-*Aspergillus fumigatus* interactions identified in different models of combined infection. The figure summarises the type of interaction and the main effects observed in published studies to-date, which are divided based on the level of complexity of the model. In vitro studies have detected predominantly antagonistic interactions of one pathogen on the other (red arrow), but a few have also described synergistic interactions (green arrows). All other models have detected increased (black arrows) pathogen burden and/or mortality and clinical studies have detected decreased lung function.

We believe that the variety of findings described above (Figure 1) are a product of the broad range of models used thus far to assess *A. fumigatus-P. aeruginosa* interactions. Hence, this field requires further and more focused investigations to gain a more complete and accurate understanding of in vivo interactions. In the future, employing experimental models such as air–liquid interface culture of human CF bronchial epithelial cells, with isogenic CF cell lines carrying specific mutations, CF transgenic mice or primary lung cells from patients with known CFTR mutations will improve the representation of the unique environment in which *A. fumigatus* and *P. aeruginosa* encounter each other during combined infection of the CF lung. Indeed, the complexity of the CF lung environment will require careful consideration during experimental design. For example, CF is characterised by progressive pulmonary deterioration and it must therefore be acknowledged that the lung environment colonised by *A. fumigatus* and *P. aeruginosa* changes over the disease course. In addition, antimicrobial treatment prescribed to CF patients is now known to impact not only the target organism, but the microbial community as a whole, which has important implications in the context of combined infection [129]. For example, Baxter et al. (2013) reported that treating respiratory exacerbations in CF patients with short-term courses of anti-*Pseudomonal* drugs reduced *Aspergillus* species abundance, indicating an inter-dependent relationship between the two species [115]. Furthermore, it is well established that *P. aeruginosa* undergoes micro-evolutions within the CF lung to adapt to the challenging environment [88] and these resultant strains seem to interact differently with *A. fumigatus* [142,148]. 

Upon consideration of all the findings, our interpretation is that the nature of *A. fumigatus-P. aeruginosa* interactions is variable, and likely dependent upon environmental factors relating to disease progression and treatment. Similarly, Zhao et al. (2018) suggested that interactions between *A. fumigatus* and *P. aeruginosa* might shift from a synergistic growth pattern, to mutual inhibition as CF lung disease progresses [120]. Initially, *P. aeruginosa* colonisation and growth as biofilm gives rise to nutritional and immunological conditions that promote concurrent growth of *A. fumigatus*. For an undefined period, the two microorganisms grow in a synergistic manner, although competition for biological resources may shift the balance towards mutual inhibition, which can still have deleterious consequences for the host due to the production of toxic molecules [120].

Overall, the diversity of microbial life in CF airways is now appreciated; however, the understanding of the dynamic nature of the lung microbiome and environment remains incomplete. At a cellular level, *A. fumigatus*-*P. aeruginosa* interactions inside the CF lung appear highly complex and are regulated by numerous external environmental factors. Determining the exact type of interactions that occur in each phase of disease will be fundamental to devise novel and/or optimised treatment strategies that can help to maintain lung function. Progressive loss of lung function caused by repeated pulmonary infections is the major cause of death in CF patients [149,150]. Therefore, if further research were to confirm that pathogen growth is enhanced in combined infection, combinatorial treatment with drugs that have a synergistic or dual effect would be indicated. In this respect, some new azoles have been described to have anti-*P. aeruginosa* activity [151] and some antibacterial drugs have direct antifungal activity [152,153] and/or synergise with common antifungals [154,155,156,157]. In contrast, if the major effect of the interaction were to trigger secretion of a toxic compound, a virulence blocker would be beneficial. For example, inhibitors of the *P. aeruginosa* elastase virulence factor, which as mentioned above is upregulated in the presence of *A. fumigatus* [145], have been reported [158,159,160].

In conclusion, even if our understanding of the interaction between *P. aeruginosa* and *A. fumigatus* has advanced significantly in the last few years, further studies employing more physiological models that factor in the complexity of the CF lung are needed to determine the exact interaction that these two pathogens undergo during combined infection. Such accurately modelling for combined infection has proved challenging thus far.

## Figures and Tables

**Table 1:** Classification of mutations in the cystic fibrosis transmembrane conductance regulator (CFTR) gene.

	CFTR Mutation Class					
	I	II	III	IV	V	VI
CFTR Defect	No functional CFTR protein	CFTR trafficking defect	Defective channel regulation	Decreased channel conductance	Reduced CFTR synthesis	Decreased CFTR stability
Mutation Type	Nonsense; frameshift; canonical splice	Missense; amino acid deletion	Missense; amino acid change	Missense; amino acid change	Splicing defect; missense	Missense; amino acid change
Examples of Causative Mutation	G542X	ΔF508	G551D	R117H	3849 + 10kbC→T	4326delTC
	W1282X	N1303K	D178R	R347P	2789 + 5G→A	G1412X
